# Action against inequalities: a synthesis of social justice & equity, diversity, inclusion frameworks

**DOI:** 10.1186/s12939-024-02141-3

**Published:** 2024-05-23

**Authors:** John C. Hayvon

**Affiliations:** Edmonton, Canada

**Keywords:** Social determinants of health, Marginalization, Intersectionality, Representation, Ethics, Action research, Participation, Practice, Community, Theories models & frameworks

## Abstract

Inequalities in health have long been recognized as interconnected with social, economic, and various other inequalities. The application of social justice and equity, diversity, inclusion (EDI) frameworks may help expand interdisciplinary perspectives in addressing inequalities. This review study conducted an environmental scan for existing syntheses of theories, models, and frameworks (TMFs) relevant to the social justice and EDI. Results from Web of Science, Scopus, PubMed, CINAHL, PsychINFO, and MEDLINE retrieved an existing implementation science framework intently centered upon health inequalities, and draws from a synthesis of postcolonial theory, reflexivity, intersectionality, structural violence, and governance theory. Given this high degree of relevance to the objective of this review, the framework was selected as a basis for expanded synthesis. Subsequent processes sought to identify social justice TMFs which could be integrated into the base framework selected, as well as to refine scope of the study. Based upon considerations of level of evidence and non-tokenistic integration, the following social justice and EDI TMFs were identified: John Rawls’ theory of justice; Amartya Sen’s Capabilities Approach; Iris Marion Young’s theories of justice; Paulo Freire’s critical consciousness; and critical race theory (CRT). The focus of the synthesis performed was scoped towards minimizing potential harms arising from actions intending to reduce inequalities. EDI considerations were not collated into a singular construct, but rather extended as a separate component assessing inequitable distribution of risks and benefits given population heterogeneity. Reflexive analysis amended the framework with two key decisions: first, the integration of environmental justice into a single construct, which helps to inform Rawls’ and Sen’s TMFs; second, a temporal element of sequential-analysis was employed over a unified output. The result of synthesis consists of a three-component framework which: (1) presents sixteen constructs drawn from selected TMFs, to consider various harms or potential reinforcement of existing inequalities; (2) aims to de-invisibilize marginalized groups who are noted to experience inequitable outcomes, and acknowledges the presence of individuals belonging to multiple groups; and (3) synthesizes seven considerations related to equitable dissemination and evaluation as drawn from TMFs, separated for sequential analysis after assessment of harms.

**Supplementary Information:**

The online version contains supplementary material available at 10.1186/s12939-024-02141-3.

## Key messages

– Synthesis of social justice and EDI theories, models, and frameworks reveals that actions - including interventions, strategies, and research itself - may present inequitable distribution of benefits and risks to different groups.

– Under existing TMFs, actions intended to reduce inequalities may still inadvertently reinforce or create inequalities. This informs the objective of *minimization of harm* for this synthesis.

– A three-component framework builds upon existing synthesis of social justice and EDI TMFs, to guide considerations of minimizing harm and equitable impact.

## Context

Existing inequalities in society are often interrelated [1–3], with scholarship documenting the value of holistic [4], interdisciplinary [5], systems-thinking [6] approaches. Exploration of the differences in terminology between *inequalities* and *inequities* indicates that certain inequalities are inseparable from scholarly notions of *social justice* [7]. The definition of social justice in itself, however, may also differ across disciplines [8, 9]. Given the interconnected nature of inequalities, multidisciplinary views on social justice may assist in holistic approaches to addressing inequalities [10]. Drawing from the body of literature in health inequalities as an example, social justice concepts have been applied in diverse projects such as: donor relations [11]; indigenous postsecondary education [12]; rural participation [13]; chronic pain management [14]; indigenous health [15]; and suicide prevention [16].

Similarly, Equity, Diversity, and Inclusion (EDI) models have emerged, with evolving and multidisciplinary definitions [17]. The relevance of EDI to actions against inequalities can be seen in its application to various disciplines, including: medical education [18]; child welfare [19]; sports [20]; climate change [21]; and emergency services [22] among others. Whether or not social justice and EDI (1) constitute separate ideologies; (2) *must* constitute separate ideologies; or (3) can be synthesized towards an integrative framework [23] forms the basis of this study.

*Action* towards the reduction of interdisciplinary inequalities may take various forms, and be interconnected to terminology including: *intervention* [24]; *implementation* [25]; *practice* [26]; *solution* [27]; *process* [28]; or *strategy* [29]. Notably, research in itself can be action [30] towards addressing social issues. The rise of participatory action research not only conceptualizes research as action - but also explicitly ties this action to the reduction of inequalities [31, 32]. Drawing from the body of evidence on participatory action research, the operational definition of *action* in this study includes research; this study does not attempt to exclude any forms of action [33], nor propose a hierarchical relation between existing forms of actions and solutions.

## Rationale & objective

The rationale for conducting this study is to acknowledge the (1) existing quantity of evidence and (2) existing synthesized theories, models, and frameworks (TMFs) relevant to social justice, towards identifying convergences for a simplified tool. Given the extensive nature of social justice TMFs, the study does not attempt systematic analysis. This article follows the reporting structure of seventeen (out of twenty-two) PRISMA-ScR guidelines. The objective is to perform synthesis of *frameworks* [34, 35] on existing social justice and EDI TMFs, with emphasis on supporting action to reduce inequalities.

## Methods

This study draws from principles guiding methods of narrative and scoping review methodologies [36], towards identifying an integrated framework of social justice and EDI. Peer-reviewed journal articles published in the English language, up to December 1st, 2023 forms the basis of eligibility criteria for inclusion. No dissertations nor conference papers were considered for this TMF study. Databases used include: Web of Science, Scopus, PubMed, CINAHL, PsychINFO, and MEDLINE. The date of the most recent search is December 1st, 2023, and no contact with authors was performed given available access to full-texts. The search strategy was adapted for fit with database functions, with four categories of search terms (Table 1). Category I search terms are designed to capture sources with relevant *thematic content*, with emphasis on the term “social justice” as it appears in its integral form. Notably, concepts relevant to EDI appear in varying arrangements in the scholarship [17, 37, 38]. In conjunction with considerable scholarship under which *equity* and *inequality* have scientific usage different from this study, application of the AND operator over the OR operator has shown utility to identify relevant sources. Category II search terms attempt to capture sources with desired structure; articles without a TMF structure were excluded from this synthesis. Category III search terms are employed to supplement the search, in a scenario where sources do not directly apply searchable terminology of TMFs. Plural forms of search terms are intentionally utilized in Category III to capture sources with an existing level of synthesis. Lastly, Category IV is employed in some databases in the *keywords*, *title*, or *abstract* fields to aid in identifying synthesized sources. Retrieval of reviews was conducted with built-in database functions where available. Category IV was not further expanded in terms of specificity (i.e. targeting of highly-synthesized sources such as *umbrella reviews* or *systematic reviews*) considering the fact that scholarship on theories may not always employ systematic review as methodology.

**Table 1:** Categories of search terms

Category I	“Social justice”; inequalit*; inequit*; equity; equality; diversity; inclusion
Category II	theor*; model; framework
Category III	Paradigm OR guidelines OR principles OR recommendations
Category IV	Review

The results of the environmental scan identified one specific source with significant potential to inform this study. Snell-Rood et al. [39] conducted a synthesis of TMFs to guide action against inequalities, with specific multidisciplinary focus on implementation science and medical anthropology. The problem statement of Snell-Rood et al. reads: “*While implementation science is driven by theory, most implementation science theories, models, and frameworks (TMF) do not address issues of power, inequality, and reflexivity that are pivotal to achieving health equity*”. The paper integrates five distinct theories: *postcolonial theory* [40]; *reflexivity* [41]; *structural violence* [42]; *intersectionality* [43]; and *governance* [44] into three separate resulting frameworks.

Given the study’s considerable alignment in objective with (1) action; (2) framework synthesis; and (3) social justice & EDI, the paper was selected as a base framework to guide further investigations in synthesis. The full-text of the paper was employed to define apt scope for extended synthesis. Four key methodological considerations and limitations emerged during this process, hereon noted as numbered *guiding principles* for the selection process of sources.

1. *Saturation, Theoretical* [45]: in assessing which other theories beyond postcolonial theory, reflexivity, structural violence, intersectionality, and governance can be synthesized, existing state of research suggests challenges in comprehensive and equitable input from all existing theories. This study is limited by its narrow-scope synthesis of a selection of five social justice and EDI TMFs into the base frameworks established by Snell-Rood et al. These five additional TMFs considered for synthesis are outlined in Table 2.
2. *Level of Evidence* [46]: notably, the base framework also makes explicit references to useful models and frameworks which are nested *under* its assessment of five theories. These include TMFs like ADAPT-ITT [47], the Behavior Change Wheel [48], and the Cultural Framework for Health [49]. This suggests that TMFs in themselves may represent varying degrees of integration of information, which in turn highlights a new consideration in defining the scope of this synthesis. The synthesis conducted in this review study did not attempt to identify more TMFs which could be nested under existing frameworks, but rather sought to explore TMFs comparable in level of evidence to the five macro-level theories considered by Snell-Rood et al. in the base framework.
3. *Synthesis* vs. *Tokenism* [50, 51]: the study seeks to acknowledge the risk of tokenist inclusion, in which each body of ideas is *listed*, but not integrated with one another. Attempts to avoid the risk of this listing led to a base-level principle to guide TMF-selection: TMFs should present sufficient overlap with more than one construct with other selected TMFs - including the five previously synthesized in the base framework - as opposed to being confined in its own construct.
4. *Providing Scope to ‘Action’*: drawing from the base framework, this synthesis seeks to acknowledge the fact that actions intending to address inequalities may present unintended, negative consequences which exacerbate inequalities for certain groups. Snell-Rood et al.’s explicit integration of reflexivity as one of the five TMFs underscores a guiding principle: actions to reduce inequality should not be automatically exempt from reflection on their inequitable impacts. This study adopts this principle in considering the scope of the output of synthesis, and aims to support the *minimization of inequitable harms* which may inadvertently accompany action against inequalities.

Data items retrieved within the sources include key principles and concepts relevant to social justice, emphasizing those which reinforce or show convergence with the base framework. Critical appraisal of individual sources was performed with the four guiding principles above. Synthesis of results was performed through a three-step process. First, key principles and concepts of each selected TMF were identified (Table 2). Second, additional principles and concepts were sought through identifying keyword convergence with the base framework. Third, any keyword convergence *between* TFMs was conducted, to assess any new perspectives emerging independently from the base framework.

Drawing upon the environmental scan, the base framework, and an integrative source on social justice theories [52], the following five TMFs were selected for synthesis of constructs:

**Table 2:** Five theories for synthesis with Snell-Rood et al., 2021, with an additional amendment

Theory	Sample Concept	Discipline
Rawls [53]	Difference Principle	Political Science
Sen [54]	Capabilities Approach	Economics
Young [55]	Five faces of oppression	Feminist Studies
Freire [56]	Emancipation	Education
Critical Race Theory [57]	Interest Convergence	Race Studies
*Environmental Justice* [58]	*“Inequitable distribution of hazards, including congenital risks”*	*Environmental Studies*

Characteristics of the sources of evidence are as follows: (1) *Date range*: compared to the TMFs excluded in this study, the six TMFs listed above are generally published at an earlier point in time; (2) *Body of Literature*: the selected TMFs tend to generate diverse, multidisciplinary perspectives from various researchers; (3) *Toolkit-Applicability*: the TMFs selected often are not conducive to being uniformly applied as a research tool or logic model given their expansive, multi-faceted natures.

### Positionality statement

The researcher acknowledges that construct synthesis and extraction is in itself influenced by the researcher’s own biases, beliefs, and contexts [59]. To make explicit these confounders, the researcher acknowledges their position [60] as a racial minority and a second-generation immigrant to a colonial territory. The researcher also acknowledges that *positions* and *identifications* can be shifting, with two key transitions upon reflection. First, the researcher did not identify with other people living with disability until later adolescence, when multiple pneumothoraces revealed a congenital defect leading to chronic condition. Second, the researcher has identified with a different ethnicity until coming to learn their familial name is translated as *barbarian* - or more accurately *ancient flesh* - in their mother tongue.

The researcher’s bias impacts analysis of construct-overlap between TMFs. To illustrate the impacts, the TMF of critical animal studies can be of value. Critical animal studies has notable overlap with social justice [61, 62], and is particularly pertinent to health research on environmental health [63] and One Health [64]. At the same time, scholarship exists on critical animal studies in relation to border studies [65], emphasizing links with marginalized, displaced, and indigenous experiences. The data-extraction process, however, would not result in synthesis beyond tokenist listing of critical animal considerations in its own construct. This exclusion of critical animal study as a TMF for synthesis, along with other social justice TMFs does not suggest lack of validity to inform social justice and EDI; rather, the limited scope of this synthesis is based upon positionality as well as the growing and evolving natures of new disciplines. Future investigation on TMFs which were not included in this synthesis will be of significant value.

The methods employed for this study include a final reflection on the synthesis, resulting in one key amendment. Similar to other TMFs which were not included for this synthesis, *environmental justice* [66, 67] as a TMF was not included based on guiding principle number three. Further analysis of the Rawlsian theory of social justice, however, reverted this decision as a key environmental justice principle was found to impact foundational construction of inequalities. This resulted in construct number 16 in the final synthesis.

Reflexive practice [68] also led to questioning of the original intent of synthesis. Although the *equality* dimension of EDI could be sufficiently captured in social justice frameworks, diversity and inclusion of various groups in society would inhabit its own construct to contradict guiding principle number three. In conjunction with the aim of this synthesis, the resulting framework was divided into three chronological segments.

## Results

The following section will first outline key concepts and learnings from the five selected TMFs. A synthesized framework emphasizing the common principles and constructs emerging from these TMFs are presented in Tables 3, 4 and 5. How each selected TMF connects to the constructs are first identified numerically, then outlined in Appendix 1. Notably, one additional construct beyond the original fifteen was integrated from environmental justice theory.

**Table 3:** Component one of three: framework to guide minimizing harms in action & research

1	Does it intentionally or unintentionally benefit a specific government, business, or industry towards greater wealth or influence?
2	Does it imply the pursuit of social justice (or reduction of inequalities) as second in priority to another objective?
3	Given existing transaction-based social structures, does it wrongly imply that social justice is always achievable without paying any price?
4	Does it prioritize social justice and emancipation for the *self* - to background or deprioritize social justice for classes, groups, and the collective marginalized?
5	Does it offer validity and visibility to the marginalized - but exclude the multiply-marginalized who live with two or more statuses of marginalization?
6	Does it dehumanize humans as machines or subordinates - by suggesting humans should accept imposition from specific individuals, institutions, ideologies, or tasks?
7	Does it distract from, conceal, simplify, or trivialize historic and systemic inequalities?
8	Does it normalize - or refuse to challenge - systemic structures under which inequalities have been allowed to persist?
9	Does it distract from, conceal, simplify, or trivialize the fact that individuals who benefit from inequalities exist, and thus can play a role in the existing landscape of inequalities?
10	Does it normalize or even glamorize accrual of influence and wealth as valid or utmost accomplishments in life?
11	Does it reinforce individuals’ tendency to consciously or subconsciously attribute legitimacy to individuals/entities holding more power and privilege (influence; wealth; etc.)?
12	Given finite resources in society, does it deprioritize the value of refusing to pursue influence & wealth towards inequalities-reduction?
13	Given the fact that basic livelihood is not guaranteed for many in society, does it deprioritize the value of sharing or dispersing wealth to the marginalized?
14	Given existing contexts in which actionable leverage points in society are mostly accessible to those with existing wealth and influence, does it sideline the marginalized by proposing actions beyond their reach - without intent to improve their access to leverage points?
15	Does it fail to acknowledge and explicitly circumvent risks of social justice which does not result in actual, on-the-ground reduction of inequalities -such as *performative* social justice?
16	Does it present some inequalities as naturally-occuring - ignoring considerable evidence on man-made environmental inequalities impacting human development in the womb?

**Table 4:** Component two of three: matrix analysis of differential benefits, risks, and perspectives of marginalized groups

	Analysis of Sixteen Constructs to Guide Minimization of Harm, Component One															
Individuals Marginalized by:	1	2	3	4	5	6	7	8	9	10	11	12	13	14	15	16
**Multiple Statuses**: ***(tally statuses below)*** [115–119]																
Poverty [120, 121]																
Intellectual Disability [122, 123]																
Physical Disability [124, 125]																
Chronic Health & Chronic Mental Health [126, 127]																
Addiction, including Neonatal Abstinence [128, 129]																
Lifelong Caregiver [130, 131]																
Foster Experience [132, 133]																
Indigenous/ Aboriginal [134, 135]																
Survivor of Abuse [136, 137]																
Victim of Crime [138, 139]																
Incarceration [140, 141]																
Homelessness or Street/Shelter Experience [142, 143]																
Race [144, 145]																
Rural or Isolated Geography [146, 147]																
Ageism: Children & Seniors [148]																
Gender [149, 150]																

**Table 5:** Component three of three: considerations of dissemination & evaluation

1	Is it both freely and logistically accessible to individuals who experience up to sixteen marginalization outlined in Component Two? Who has been successfully sampled or engaged, and what is the quantitative average of simultaneous marginalizations experienced by the sample?
2	Given existing transaction-based structures, does it take into account *opportunity costs* in engagement - in terms of participants’ time, labor, health, mental well-being, relationships etc. - especially for individuals who lack access to basic livelihood?
3	If it promises or implies certain outcomes, is it explicit in communicating the actual risks of such outcomes not being successfully attained?
4	During engagement, does it actively acknowledge and address how the marginalized may be forced - or taught - to self-censor AND align with privileged perspectives?
5	Does it analyze in-depth what positive sentiments or positive feedback is based upon?
6	Does it acknowledge and intently explore simultaneous and inadvertent negative impacts alongside the positive?
7	Does it acknowledge the dynamic, shifting nature of actions as processes, and address negative impacts which may arise at future points in time despite previous positive assessment?

With regards to critical appraisal within sources of evidence, the synthesis did not attempt to comprehensively capture all concepts within the five TMFs above, but rather emphasizes principles converging with the base framework selected.

### Results of individual sources of evidence

*Rawls* (1971). Rawlsian theory is one of the earliest frameworks explicitly using the phrase *social justice*, and is notably philosophical in its nature [69]. In Rawls’ own words, the TMF is intended as a product “which generalizes and carries to a higher level of abstraction”. Notably, Rawlsian theory exerts significant influence over new generations of general social justice TMFs, including the second core TMF synthesized [70, 71]. As a general theory, this TMF does not concern itself exclusively with the experiences of marginalized groups nor individuals experiencing social injustices. Political considerations of liberties, rights, responsibilities, and resources in relation to society as a whole form the basis of conceptualizing inequalities. Of note, the Difference Principle [72] in Rawlsian theory has considerable *implicit* overlap with intersectionality, one of the original TMFs synthesized by the base framework. Rawlsian theory proposes that ideally social inequalities “are to be to the greatest benefit of the least-advantaged members of society” [53]. This emphasis on benefiting individuals who may be considered least-advantaged given multiple, simultaneous, and mutually-reinforcing statuses of marginalization emerges as present in early conceptualization of social justice. Overall, Rawlsian contemplation of the role of institutions; profit; competition for livelihood; justice as first virtue; influence; and society as an integrated unit informs the synthesis of constructs number 1, 2, 4, 8, 9, 12, 13 Table 3).

*Sen* (1974). Existing scholarship mostly refers to this TMF as the Capabilities Approach [73], which has been a dominant TMF influencing social justice in relation to global development [74]. The TMF is the result of Sen’s reflection of Rawlsian theory, and employs considerable positive conceptualizations such as: freedom, well-being, choice, and human-potential. The outcome-focused orientation of this TMF highlights the fact that fair distribution of resources - as means - may not be sufficient to achieve equitable ends, since marginalized individuals often are forced to bear greater costs and risks in order to achieve the same life opportunities [54]. Within this construction, there is intentional interrogation of the actual value of material resources to address existing inequalities. Further, the concept of freedom as positioned in this TMF provides grounds for analyzing factors which deprive individuals from personal choice-making in inequitable circumstances [75]. Lack of agency is interconnected with survival and basic livelihood, suggesting there is a “cut-off point” which warrants a targeted lens on deprivation [76]. In relation to this synthesis, the Capabilities Approach informs constructs number 2, 3, 6, 10, 12, 14.

*Young* (1990). Iris Marion Young’s feminist theories are interconnected with her social justice theories without exclusive focus on gender [77, 78]. Young’s theories on social justice may not be easily considered as a singular TMF, but instead takes the form of multiple TMFs presented as a body of work. Three key components are drawn upon for this synthesis study. First of all, the Politics of Difference is emphatic in its consideration of social groups, as opposed to Rawls’ and Sen’s philosophy of all individuals in society [79]. Young’s theory, in turn, highlights heterogeneity between individuals in relation to shared historic or systemic experiences of inequalities. This lens helps consider whether identical actions to reduce inequalities will have the same efficacy and impacts on various groups in society [80]. In conjunction with the Politics of Difference, Young’s Social Connection Model proposes that the same structure which creates inequalities for certain groups can simultaneously advance the opportunities and influence of other groups [81]. This highlighting of structural inequalities is complemented by Young’s argument for Five Faces of Oppression, which categorizes inequalities into: economic exploitation, socio-economic marginalization, powerlessness over one’s work, cultural imperialism, and systematic violence [55]. In combination, Young’s theories of social justice informs the synthesis of constructs number 1, 3, 5, 6, 7, 8.

*Freire* (1968). While the overlap between Freirean theory and social justice is primarily studied in the field of social justice pedagogy, Freirean theory has found various applications beyond pedagogical settings [82, 83]. This TMF positions inequalities as stemming from the objective of maximizing possessions in society, and perpetuated by the context in which marginalized groups are led to aspire to this objective [56]. Another key concept in the Freirean TMF is *critical consciousness*, which serves to identify an additional cause of inequalities: under Freire’s conceptualization, individuals experiencing inequalities often do not have access to knowledge which gives them vision of inequalities, and are thus further barred from opportunities to act as transformers of an inequitable society [84]. *Action* is positioned as central to this TMF, as Freire further expands the concept of critical consciousness into three elements of *critical reflection, political self-efficacy*, and *critical action* [85, 86]. Freirean theory suggests that individuals who experience inequalities can reduce inequalities when they take action [87], which in turn rests upon a pedagogy which supports this action. In total, this TMF informs the synthesis of constructs number 2, 4, 10, 11, 12, 15.

*Critical Race Theory*. Various key scholars can be identified with this body of research, and in comparison to the four aforementioned TMFs, Critical Race Theory should be acknowledged as an evolving discipline with new concepts being constructed [88, 89]. It should be noted that *intersectionality* [43, 90] - a key concept in Critical Race Theory - has been employed in Snell-Rood et al. in establishing the base framework informing this synthesis. Two additional concepts were drawn upon to inform this study. First, the Social Construction of Race [91] highlights the fact that individuals and groups can collectively construct ideologies which lead to inequalities. Under this TMF, action towards reducing inequalities may consider sociocultural dimensions to explore leverage points - echoing scholarship in queer studies [92] and critical disability theory [93, 94]. Social Construction of Race also considers how individuals construct privilege for certain groups, often for groups that have access to being perceived as *normative* [95]. Second, Interest Convergence reinforces aforementioned TMFs in informing action against inequalities. Under Critical Race Theory, action against inequalities emerges often as unsuccessful unless it aligns with the interests of those who do not experience inequalities [96]. Critical Race Theory as a whole echoes Young’s TMFs on social justice, highlighting the experiences of groups as well as the role of systems and structures [97]. Additionally, it expands the lens of the Freirean TMF on action, to consider how even when individuals have access to knowledge and leverage points towards reducing inequalities, additional actors in society have capacity to approve of these actions [39]. Beyond providing perspective on specific races and ethnicities experiencing inequalities, Critical Race Theory informs constructs number 4, 5, 7, 8, 9, 11 for this synthesis.

*Environmental Justice*. This synthesis did not attempt comprehensive integration of Environmental Justice as a TMF, but notes its importance in informing the TMFs proposed by Rawls and Sen. Both TMFs explicitly consider the role of what may be perceived as naturally-occuring inequalities, such as disabilities occurring from birth. These inequalities historically may have been used to justify a passive stance on reducing inequalities, as inequalities are seen as both a given and as beyond the actionable leverage points of humanity. Developments in environmental health, however, have amassed a strong body of evidence [98–102] on how seemingly naturally-occuring inequalities such as educational achievement [103]; IQ [104]; emotional stability [105]; life expectancy [106]; personality traits [107]; tendencies towards criminal behavior [108]; and biological vulnerability to substance addiction [109] can be determined by environmental pollutants in the womb. The neural development of the fetus in early stages emerges as an intricate process under the influence of human pollution and intervention. Notably, these pollutants have capacity to also influence children in their early developmental stages, as well as adults [110, 111]. As certain inequalities were embraced as natural phenomena prior to the scientific discovery of such linkages, this TMF highlights the potential of research to explore validity of attempts to justify inequalities. This specific link to expand upon Rawls’ and Sen’s TMF is documented in construct number 16 of the synthesis.

### Synthesis of results

Synthesis of concepts was performed by open coding, with codes drawn from the base framework. Axial coding was performed to identify convergence between themes, particularly emphasizing recurring themes. Appendix 1 provides a snapshot of the coding process, through emphasizing key principles in the form of quotes. Notably, not all relevant quotes could be encapsulated in the table. Contextuality is limited - as quotes which have the highest keyword-density are prioritized for reasons of length and legibility of the table. Quotes which were not drawn upon to directly inform a construct, but helped to add nuanced analysis of a construct are shown with an asterisk. As noted, the selected overarching TMFs are expansive and lead to multi-faceted academic discourse. Where possible, quotations from the original author (with the exception of Critical Race Theory, which refers to the works of multiple authors) are prioritized - while acknowledging that varying interpretations of these TMFs can exist across scholarship.

### Synthesized framework

The summary of evidence is presented in the form of three components. First, sixteen constructs of social justice are outlined (Table 3). Second, data-extraction and the environmental scan compiled groups experiencing inequalities, in order to capture the diversity and inclusion of various marginalized individuals (Table 4). In conjunction, the first two components of the synthesis aim to guide considerations of minimizing harms of actions, including actions intended to reduce inequalities. The third component aims to present a temporal element to the synthesis, as it intently focuses on dissemination and evaluation post-action.

The constructs above may be assessed from the perspectives of multiple marginalized groups. The diversity and inclusion of specific groups in a social justice framework was conceptualized in this synthesis as follows: drawing upon Young, Freire, and Snell-Rood et al’s shared construct, EDI emerges as too complex [112–114] to be confined to a singular construct. Instead, each of the sixteen constructs has capacity to present differing - or, inequitable - levels of benefits and risks depending on the sub-group of focus. Component Two of the framework (Table 4) aims to explicitly promote the inclusion of sixteen diverse groups which may be challenging to equitably, meaningfully, and respectfully engage in action and research. The structure of this second component emerges as a matrix together with Component One, to intently explore heterogeneity and diversity of impacts.

The first column of the matrix highlights the possibility that individuals can live with none or up to sixteen simultaneous marginalizations. Theoretical questions emerge, such as whether sixteen individuals each representing a marginalization can effectively represent the perspectives of a single individual with sixteen simultaneous marginalizations [151]. Field testing of the synthesized framework will bring significant value to its applicability.

The third component of the framework presents seven considerations which chronologically follow Component One and Component Two. In combination, the synthesis builds upon Snell-Rood et al’s integration of five TMFs, towards an objective of supporting future actions against inequalities. This specific synthesis output emphasizes the minimization of harms and risks which may arise with actions - as informed by the base framework’s positionality in implementation science. Existing scholarship highlights the fact that actions intending to reduce inequalities may inadvertently pose inequitable distributions of harms and risks, particularly when diverse individuals are included in analysis. The explicit inclusion of sixteen such groups in this synthesis aims to (1) highlight the fact that individuals may experience numerous marginalizations simultaneously, and (2) expand EDI as a complex consideration.

### Limitations

This review is limited by two key research directions: first, the expansion of an existing framework as basis, and second, the selection of social justice frameworks in a non-comprehensive manner guided by the base framework. The results lean heavily towards a narrative summary as opposed to systematic review methodology. Selection of TMFs to be integrated into the base framework is impacted by existing synergies in addition to matching levels of TMFs as presented in the base framework. As present in Snell-Rood et al.’s categorization of theories, five overarching TMFs (i.e. *postcolonial theory; reflexivity; structural violence; intersectionality; governance*) are contrasted against TMFs as tools, such as ADAPT-ITT. This synthesis is limited in its exclusion of all TMFs which would likely be nested under Snell-Rood’s overarching TMFs.

This synthesis study is limited by its emphasis on peer-reviewed sources in the English language. The base framework and its incorporation of postcolonial theory suggests value in exploring varying paradigms, worldviews, and traditional teachings [6, 152, 153] in guiding social justice action. While postcolonial theory informs the base framework for this synthesis, this study is notably limited in its engagement with traditional knowledge of various indigenous cultures around the world.

Despite efforts to acknowledge the interconnectedness of various concepts related to inequalities, this synthesis exclusively considers TMFs that focus on social justice. In short, TMFs which cannot be captured by the search term of “social justice” are not adequately considered in this synthesis. Other relevant terminology and fields of research will add value to providing a holistic perspective on action against inequalities.

As a review study, this study is limited in its procedure for quality assessment of included TMFs. This study did not attempt to evaluate the validity or rigor of TMFs relevant to social justice; guiding considerations for selection of TMFs were determined by the level of evidence as well as potential overlap with the selected base framework.

The synthesis process was guided by an existing framework which sought to integrate equity-based TMFs towards informing *implementation science*. As such, future studies which elect to expand on another previously-conducted synthesis may achieve widely-diverging results. The base framework selected for this synthesis draws from postcolonial theory, reflexivity, structural violence, intersectionality, and governance theory. After this synthesis process, the social justice TMFs incorporated in this synthesis cannot be viewed as comprehensively represented in their wholeness. TMFs are notably selected to inform the pre-established objective of minimizing harms, or, minimizing risks of exacerbating inequalities.

This synthesis was guided by a data extraction process emphasizing overlaps between five theories to be synthesized, and with the base framework. No exploration of *diverging* perspectives was performed, and these perspectives were not included in the final synthesis.

### Potential application

With growing emphasis on action and implementation of research towards the reduction of inequalities, the framework seeks to integrate existing peer-reviewed TMFs relevant to social justice. The anticipated application is largely scoped towards: (1) minimizing inadvertent detriments to health equity which may simultaneously emerge alongside benefits, and (2) inclusion of intersectionally-marginalized participants in assessing inequitable impacts arising from action. The framework seeks to support utility via identifying converging perspectives in a simplified form, as quantity of existing theories may present challenges for integrated application.

## Conclusions

This study builds upon an existing synthesis of equity-related TMFs to guide implementation science in addressing health inequalities. Overlapping concepts between Rawls, Sen, Young, Freire, and Critical Race Theory are drawn upon to explore a synthesis of social justice and EDI considerations. The synthesis process is guided by the objective of minimizing harms and risks of certain actions - including those with the intent of reducing inequalities. The resulting product is a three-component framework outlining various considerations of harms and risks, to be evaluated by diverse individuals experiencing up to sixteen marginalized statuses as represented in the second component of the framework. Equality in dissemination and evaluation are separated into a subsequent third component.

Social justice and EDI concepts in existing literature share sufficient overlap to facilitate attempts at synthesis. At the same time, the complexity of the evidence base suggests that multiple syntheses can be conducted to meet differing *objectives*. In particular, this framework only attempts to consider minimization of harms and risks associated with actions. Future investigations which (1) meet the diverse needs of TMF-users; (2) explore social justice TMFs excluded in this synthesis; or (3) adopt a different base framework will all contribute greatly to scholarly understanding on inequalities.

### Electronic supplementary material

Below is the link to the electronic supplementary material.

Supplementary Material 1

## Data Availability

No datasets were generated or analysed during the current study.

## Abbreviations

EDI: Equity, Diversity, and Inclusion
TMF: Theories, Models, and Frameworks
