# Supplementary material for: Action against inequalities: a synthesis of social justice & equity, diversity, inclusion frameworks
Source: Int J Equity Health. 2024 May 23;23:106. doi: 10.1186/s12939-024-02141-3 (PMC11119020; doi:10.1186/s12939-024-02141-3)
Supplement: Supplementary file 1 — Supplementary Material 1 [file 12939_2024_2141_MOESM1_ESM.docx]

|  |  | **Base Framework** | **Rawls** | **Sen** | **Young** | **Freire** | **Critical Race** |
| --- | --- | --- | --- | --- | --- | --- | --- |
| 1 | Does it intentionally or unintentionally benefit a specific government, business, or industry towards greater wealth or influence? | *“[actions can] manage populations deliberately and inadvertently exert power”* | *“ The principles of justice are chosen behind a veil of ignorance. This ensures that no one is advantaged or disadvantaged in the choice of principles.”* |  | *“social justice means the elimination of institutionalized domination and oppression”* |  |  |
| 2 | Does it imply the pursuit of social justice (or reduction of inequalities) as second in priority to another objective? | *“Failing to do so can threaten our efforts to implement interventions, invalidate our scientific findings, and, most importantly, perpetuate social and economic inequalities.”* | *“According to the difference principle, [inequality] is justifiable only if the difference in expectation is to the advantage of the representative man who is worse off,”* | *“the freedom to achieve well-being is of primary moral importance”* |  | *“This, then, is the great humanistic and historical task of the op- pressed: to liberate themselves and their oppressors as well.”* |  |
| 3 | Given existing transaction-based social structures, does it wrongly imply that social justice is always achievable without paying any price? | *“consistently failed to cover the full costs of those services, ultimately undermining their quality and availability”* |  | *“Whether someone can convert a set of means - resources and public goods - into a functioning (i.e., whether she has a particular capability) crucially depends on certain personal, sociopolitical, and environmental conditions”* | *“Structural injustice occurs as a consequence of many individuals and institutions acting in pursuit of their particular goals and interests, within given institutional rules and accepted norms.”* |  |  |
| 4 | Does it prioritize social justice and emancipation for the *self* - to background or deprioritize social justice for classes, groups, and the collective marginalized? | *“Does the intervention include unstated cultural values (e.g., model of …premised on individualism)”* | *“The basic rights and liberties and their priority are there said to guarantee equally for all citizens”* |  |  | *“The latter, by distorting the authentic relation between the Subject and objective reality, also separates the cognitive, the affective, and the active aspects of the total, indivisible personality.”* | *“as long as women are using class or race power to dominate other women, feminist sisterhood cannot be fully realized.” (bell hooks)* |
| 5 | Does it offer validity and visibility to the marginalized - but exclude the multiply-marginalized who live with two or more statuses of marginalization? | *“violent social structures intersect, exposing some populations (e.g., sexual and gender minorities of color) to multiple forms of discrimination”* |  |  | *“forms of communication that women and people of color tend to use, including, as she puts it, “greeting, rhetoric, and storytelling”. Young argued that these alternative modes of communication could provide the basis of a more democratic, communicative theory.”* |  | *"Intersectionality is a metaphor for understanding the ways that multiple forms of inequality or disadvantage sometimes compound themselves and create obstacles that often are not understood among conventional ways of thinking." (Kimberlé Williams Crenshaw)* |
| 6 | Does it dehumanize humans as machines or subordinates - by suggesting humans should accept imposition from specific individuals, institutions, ideologies, or tasks? | *“Decision-making largely reverted to people already in positions of power…”* |  | *“people living in deprivation may actually tend to lower their expectations as a coping mechanism. But just because they experience a bigger increase in well-being from fewer resources, it does not mean that the situation is justified or justifiable in the first place.”* | *“The powerless are those who lack authority or power even in this mediated sense, those over whom power is exercised without their exercising it; the powerless are situated so that they must take orders and rarely have the right to give them.”* |  |  |
| 7 | Does it distract from, conceal, simplify, or trivialize historic and systemic inequalities? | *“scientific and medical knowledge has developed in concert with co lonialism; [action]... can further these historic relation- ships.”* |  |  | *“those who have been historically silenced have a difficult time having their views heard or heeded.”* |  | *“One of the most disturbing consequences of colonization could well be this notion of a single History…The struggle against a single History for the cross-fertilization of histories means repossessing both a true sense of one’s time and identity…” (Edouard Glissant)* |
| 8 | Does it normalize - or refuse to challenge - systemic structures under which inequalities have been allowed to persist? | *“most…theories, models, and frameworks (TMF) do not address issues of power, inequality, and reflexivity that are pivotal to achieving health equity.”* | *“ to work out a conception of justice that provides a reasonably systematic alternative to utilitarianism, which in one form or another has long dominated…thought”* |  | *”A concept of political responsibility says that we who are part of these processes should be held responsible for the structural injustice, as members of the collective that produces it, even though we cannot trace the outcome we regret to our own particular actions in a direct causal chain. A concept of political responsibility fills this role without attributing blame.”* |  | *“Capitalism and racism, in other words, did not break from the old order but rather evolved from it to produce a modern world system of "racial capitalism" dependent on slavery, violence, imperialism, and genocide.” (Cedric Robinson)* |
| 9 | Does it distract from, conceal, simplify, or trivialize the fact that individuals who benefit from inequalities exist, and thus can play a role in the existing landscape of inequalities? | *“projects…can delegitimize other vital forms of healing or may be used to control particular popula- tions and gain profit”* | *“Normally the theory of an institution, just as that of a game, takes the constitutive rules as given and analyzes the way in which power is distributed and explains how those engaged in it are likely to avail themselves of its opportunities.”* |  | *“The central insight expressed in the concept of exploitation…is that this oppression occurs through a steady process of the transfer of the results of the labor of one social group to benefit another.”* |  | *“Corporate elites used the mandate for diversity, for example, as a means to create a diverse managerial class more effective at controlling the broad multi-racial low-paid workforce.” (Derrick Bell)* |
| 10 | Does it normalize or even glamorize accrual of influence and wealth as valid or utmost accomplishments in life? |  | ** “ In buying and selling to maximize satisfaction or profits, households and firms are not giving a judgment as to what is from a social point of view the most efficient economic configuration“* | *“utilitarian measures of well-being…[is defined] in terms of the amount of utility, such as pleasure or happiness, that they derive from the resources and goods that they have”* |  | *“One of the methods of manipulation is to inoculate individuals with the bourgeois appetite for personal success.”* |  |
| 11 | Does it reinforce individuals’ tendency to consciously or subconsciously attribute legitimacy to individuals/entities holding more power and privilege (influence; wealth; etc.)? | *“sought to tap the perspectives of these stakeholders [yet]…Decision-making largely reverted to people already in positions of power”* |  |  | ** “in the process of trying to reach consensus, the untrained voices of women and others who have been marginalized would be left out of the final tally”* | *“As long as [the oppressed[ live in the duality in which to be is to be like, and to be like is to be like the oppressor”* | *“The colonized intellectual mediates the relation of the colonized for the colonizer, translating the terms of colonial life into the language, concepts, and thinkable politics of the colonial power.” (Frantz Fanon)* |
| 12 | Given finite resources in society, does it deprioritize the value of refusing to pursue influence & wealth towards inequalities-reduction? | *‘What do [agents] from resource- wealthy countries or institutions have to gain in this work and what might people in local settings stand to lose?”* | *“In each [inequitable] case there is a single person whose system of desires determines the best allocation of limited means.”* | *“the amended utilitarian position would actually direct more resources and freedoms to the able-bodied person and less to the disabled one”*  *“Food may be abundant in the village, but a starving person may have nothing to exchange for it, no legal claim on it, or no way of preventing intestinal parasites from consuming it before he or she does. In all these cases at least some resources will be available, but it will still leave that person hungry and, after a while, undernourished.”* |  | *“Discovering himself to be an oppressor may cause consider- able anguish, but it does not necessarily lead to solidarity with the oppressed. …Solidarity requires that one enter into the situation of those with whom one is solidary;”* |  |
| 13 | Given the fact that basic livelihood is not guaranteed for many in society, does it deprioritize the value of sharing or dispersing wealth to the marginalized? | *“draws attention to social and economic patterns that cause violent harm, such as institutional racism, poverty, and discrimin- ation, that expose some people disproportionately to health risks and constrain their ability to meet their own basic needs”* | *“ Equality of opportunity means an equal chance to leave the less fortunate behind in the personal quest for influence and social position. Thus a meritocratic society is a danger “* |  |  |  |  |
| 14 | Given existing contexts in which actionable leverage points in society are mostly accessible to those with existing wealth and influence, does it sideline the marginalized by proposing actions beyond their reach - without intent to improve their access to leverage points? | *“These structural factors explain not only what makes [action] successful but also why some people are so much sicker than others and why barriers to evidence-based intervention may be greater in certain delivery settings.”* | ** “This weighting, however, is normally influenced by the demands of different social interests and so by relative positions of power and influence.”*  *“There exists a marked disparity between the upper and lower classes in both means of life and the rights and privileges of organizational authority. The culture of the poorer strata is impoverished while that of the governing and technocratic elite is securely based on the service of the national ends of power and wealth.”* | *“Real freedom in this sense means that one has all the required means necessary to achieve that doing or being if one wishes to. That is, it is not merely the formal freedom to do or be something, but the substantial opportunity to achieve it.”* | **”structures refer to the relation of social positions that condition the opportunities and life prospects of the persons located in those positions.”*  *“Marginals are people the system cannot or will not use”* |  |  |
| 15 | Does it fail to acknowledge and explicitly circumvent risks of social justice which does not result in actual, on-the-ground reduction of inequalities -such as *performative* social justice? | *“purported to increase the voice of consumers by incorporating persons with lived experience of serious mental illness, their families, and representatives from community organizations”*  *“These factors are often glossed as cultural appropriateness or acceptability”* |  |  |  | *“In order to have the continued opportunity to express their "generosity," the oppressors must perpetuate injustice as well. An unjust social order is the permanent fount of this "generosity"”* |  |
| 16 | Does it present some inequalities as naturally-occuring - ignoring considerable evidence on man-made environmental inequalities impacting human development in the womb? | *(Environmental Justice)* | | | | | |

Appendix 1. Sample quotations selected to illustrate snapshot of convergence and co-refinement of constructs across selected social justice TMFs
